# The Role of an MRI-Based Radiomic Signature in Predicting Malignancy of Parotid Gland Tumors

**DOI:** 10.3390/cancers15133319

**Published:** 2023-06-23

**Authors:** Delia Doris Muntean, Sorin Marian Dudea, Mihaela Băciuț, Cristian Dinu, Sebastian Stoia, Carolina Solomon, Csutak Csaba, Georgeta Mihaela Rusu, Lavinia Manuela Lenghel

**Affiliations:** 1 Department of Radiology, Faculty of Medicine, “Iuliu Hațieganu” University of Medicine and Pharmacy, 400012 Cluj-Napoca, Romania; muntean.delia.doris@elearn.umfcluj.ro (D.D.M.); sdudea@umfcluj.ro (S.M.D.); csutakcsaba@elearn.umfcluj.ro (C.C.); mihageorgeta@elearn.umfcluj.ro (G.M.R.); pop.lavinia@umfcluj.ro (L.M.L.); 2 Department of Maxillofacial Surgery and Implantology, Faculty of Dentistry, “Iuliu Hațieganu” University of Medicine and Pharmacy, 400012 Cluj-Napoca, Romania; mbaciut@umfcluj.ro (M.B.); cristian.dinu@umfcluj.ro (C.D.); sebastian.stoia@umfcluj.ro (S.S.)

**Keywords:** radiomics, textural analysis, parotid gland tumors, differential diagnosis, MRI

## Abstract

**Simple Summary:**

Differentiating between benign and malignant parotid gland tumors is of paramount importance as it impacts therapeutical management. MRI represents the best imaging technique in diagnosing and characterizing parotid gland tumors, offering a high soft tissue contrast resolution. However, there are still overlapping radiological features between tumoral types; thus, accurate malignancy detection remains a challenge. Recently, radiomics has gained recognition as a promising new non-invasive approach in oncological imaging, especially related to tumor classification, and has the potential to become an additional diagnostic tool that might offer support in the clinical decision-making scenario.

**Abstract:**

The aim of this study was to assess the ability of MRI radiomic features to differentiate between benign parotid gland tumors (BPGT) and malignant parotid gland tumors (MPGT). This retrospective study included 93 patients who underwent MRI examinations of the head and neck region (78 patients presenting unique PGT, while 15 patients presented double PGT). A total of 108 PGT with histological confirmation were eligible for the radiomic analysis and were assigned to a training group (*n* = 83; 58 BPGT; 25 MPGT) and a testing group (*n* = 25; 16 BPGT; 9 MPGT). The radiomic features were extracted from 3D segmentations of the PGT on the T2-weighted and fat-saturated, contrast-enhanced T1-weighted images. Following feature reduction techniques, including LASSO regression analysis, a radiomic signature (RS) was built with five radiomic features. The RS presented a good diagnostic performance in differentiating between PGT, achieving an area under the curve (AUC) of 0.852 (*p* < 0.001) in the training set and 0.786 (*p* = 0.017) in the testing set. In both datasets, the RS proved to have lower values in the BPGT group as compared to MPGT group (*p* < 0.001 and *p* = 0.023, respectively). The multivariate analysis revealed that RS was independently associated with PGT malignancy, together with the ill-defined margin pattern (*p* = 0.031, *p* = 0.001, respectively). The complex model, using clinical data, MRI features and the RS, presented a higher diagnostic performance (AUC of 0.976) in comparison to the RS alone. MRI-based radiomic features could be considered potential additional imaging biomarkers able to discriminate between benign and malignant parotid gland tumors.

## 1. Introduction

Parotid gland tumors (PGT) are rare, accounting for approximately 3% of all tumors in the cervical region and 80–85% of salivary gland tumors [1]. Approximately 80% of PGT are benign, the most common being pleomorphic adenomas and Warthin’s tumors, while the remaining 20% of PGT are malignant [2,3].

The gold standard treatment for PGT is surgery, and the protocol is determined by the histopathological type of tumor: for benign lesions, local excision or partial parotidectomy may be sufficient, whereas for malignant lesions total parotidectomy and neck dissection is recommended [4].

For assessing PGT, the imaging method of choice is magnetic resonance imaging (MRI), which offers valuable information regarding morphology, disease extension, and deep surrounding structures imaging [1,5]. The main advantages of MRI over other imaging methods include the absence of ionizing radiation and an improved tissue contrast resolution which is particularly important in detecting tumoral local spread including perineural disease [6].

Multiparametric MRI allows morphological and signal pattern analysis of PGT using standard sequences (T1-WI, T2-WI, STIR). Moreover, MRI also allows for a functional assessment of PGT by using diffusion-weighted imaging with the corresponding ADC maps, and dynamic contrast-enhanced imaging, respectively [1,3]. Several algorithms have been proposed to determine the histopathological type of parotid tumors, but there is still no general validation of them as there are several overlapping MRI features between lesions [7]. An accurate differential diagnosis is mandatory to implement a clinically and surgically appropriate strategy for PGT patients.

In oncological imaging, radiomics has recently emerged as a promising novel non-invasive tool, particularly useful in areas such as tumor classification, prognosis prediction, or therapeutical response assessment [8,9]. Radiomic analysis refers to the extraction of numerous quantitative imaging features from specific regions of interest in medical images. The most discriminative radiomic features can further be combined into radiomic signatures, which can be used to differentiate between various tumors [10,11].

Although MRI-derived radiomic signatures have shown potential in distinguishing between different types of salivary gland tumors [12,13], a consistent generally validated signature has not yet been established in the literature.

The purpose of this study is to assess whether textural analysis biomarkers are able to predict malignancy in PGT using standard MRI sequences and to evaluate the diagnostic performance of the resulting predictive radiomic model. A second aim is to assess the radiomic analysis added benefit to clinical–radiological models in the differentiation between benign parotid gland tumors (BPGT) and malignant parotid gland tumors (MPGT).

## 2. Materials and Methods

This study adhered to the Declaration of Helsinki guidelines and was granted approval by the Ethical Committee of the “Iuliu Hațieganu” University of Medicine and Pharmacy Cluj-Napoca (registration number: 43; date: 11 February 2022). Owing to the retrospective nature of the research, informed consent was waived for all participants.

### 2.1. Study Population

A retrospective analysis was performed in the electronic clinical and radiological database for patients who underwent an MRI evaluation of the head and neck region during January 2018 and January 2023 to assess parotid gland lesions.

The following inclusion criteria were used: (1) patients with PG-related symptoms or masses; (2) histological confirmation of the PGT from biopsy or surgical specimen; (3) available MRI examinations with corresponding technical parameters mentioned in the “Image Acquisition” Section 2.2.

The exclusion criteria were as follows: (1) tumors with a maximum diameter <5 mm; and (2) the existence of imaging artifacts making the images unsatisfactory for radiomic analysis. The exclusion criterion for a maximum tumor diameter of 5 mm was set to reduce the potential impact of partial volume effects that could alter the genuine tissue-specific image texture [14].

A final cohort of 93 consecutive patients was formed, following the application of the inclusion and exclusion criteria. A total of 78 patients presented unique PGT, while 15 patients presented double PGT (8 patients with double Warthin tumors, 4 patients with double malignant tumors, and 3 patients with double pleomorphic adenoma).

This study included a final number of 108 PGT which were eligible for radiomic analysis. Using the “one-third” criteria proposed in radiomic studies [15], the samples were randomly divided into a training group (83 PGT; 58 benign, 25 malignant) and a testing group (25 PGT; 16 benign, 9 malignant).

### 2.2. Image Acquisition

The MRI examinations were performed in a single center, using a 1.5 Tesla MRI scanner (SIGNA™ Explorer, General Electric, Milwaukee, WI, USA) using a dedicated, 16-channel, high-resolution head coil. The acquisition protocol was constructed an of axial fast spin-eco (FSE) T1-WI; an axial FSE T2-WI using the PROPELLER (Periodically Rotated Overlapping ParallEL Lines with Enhanced Reconstruction) technique; a coronal STIR PROPELLER; an axial diffusion-weighted imaging (DWI) using echo-planar imaging sequences at multiple b-values with the corresponding ADC maps; an axial perfusion-weighted imaging; and an axial FSE fat-saturated contrast-enhanced T1-WI using intravenous contrast medium 0.1 mL/kg Gadobudrol, (Gadovist; Bayer HealthCare, Berlin, Germany).

For the textural analysis, the following sequences were used: axial FSE T2-WI PROPELLER (T2-WI) and axial fat-saturated contrast-enhanced T1-WI (fsCE-T1-WI). The corresponding MRI specifications are presented in Table 1.

### 2.3. Preprocessing, Segmentation, and Feature Extraction

Each examination was reviewed on a dedicated workstation (General Electric, Advantage workstation, 4.7 edition) by one radiologist specialized in head and neck imaging with more than 15 years-experience in MRI, who reviewed the images for possible artifacts and protocol errors. All examinations underwent anonymization, and the selected sequences were retrieved in DICOM format and imported into an open-source texture analysis software, Slicer version 4.11 (available online at: http://www.slicer.org/ accessed on 1 February 2023).

Within the 3D Slicer program, before segmentation and feature extraction, all MR images went through several preprocessing stages. Firstly, all images were normalized by division through standard deviation and the gray values were discretized using a fixed-bin width of 25. To maintain consistency in scaling and orientation while extracting 3D features, all images were resampled with a voxel size of 1 × 1 × 1 mm^3^ using a B-Spline interpolator. Isotropic resampling was preferred over anisotropic resampling as it is more suitable for computing 3D textural features [16]. The widely used “µ ± 3σ” algorithm was also utilized for image intensity normalization and for diminishing the fluctuation impact of the acquired scanning parameters of the MRI examination [17]. Therefore, image intensity outliers (which differed > 3 sigma from the mean) were identified and removed.

After the preprocessing steps, one radiology resident (DDM), blinded to the histopathological results, manually outlined each parotid lesion on sequential images; therefore, a 3D segmentation was performed, excluding vessels or areas with necrosis.

**Figure 1:**
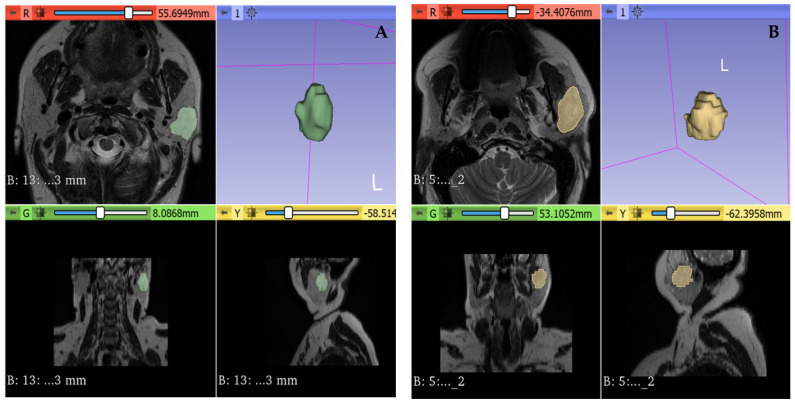
(**A**) 3D segmentation of a benign parotid tumor (confirmed as pleomorphic adenoma). (**B**) 3D segmentation of a malignant parotid tumor (confirmed as adenoid cystic carcinoma).

The contouring procedure aimed to cover the tumor’s maximum area without exceeding the lesion’s margin. Figure 1 shows an example of benign and malignant PGT 3D segmentation.

For the radiomic features extraction, the open source PyRadiomics package (version 3.0.1) was used. From each MRI sequence, 1037 quantitative radiomic features were extracted from the 3D segmentation of the PGT, using both original and filtered images.

The preprocessing filters were Laplacian of Gaussian (LoG), using fine and coarse patterns (sigma 3.0 and 5.0 mm) and wavelet filters.

The extracted radiomic features belonged to the following groups: first-order, gray-level co-occurrence matrix (GLCM), gray-level run length matrix (GLRLM), gray-level size zone matrix (GLSZM), gray-level dependence matrix (GLDM), neighboring gray-tone difference matrix (NGTDM), and shape.

### 2.4. Feature Selection and Statistical Analysis

The first step in the feature selection process was to assess feature stability between two segmentations. Therefore, 50 randomly selected PGT were resegmented by the same radiologist (DDM), two months after the initial segmentation. The intraobserver agreement was assessed by calculating the intraclass correlation coefficient (ICC). Features with an ICC ≤ 0.75 were excluded from further analysis.

All radiomic features underwent Z-score normalization.

To control data overfitting in this radiomic study, the following feature selection steps were conducted.

Firstly, to identify the statistically significant radiomic features able to differentiate between BPGT and MPGT, a univariate test was applied (Mann–Whitney U), using the Benjamini–Hochberg correction as an adjustment for multiple testing (corrected *p*-values < 0.05).

Secondly, to eliminate redundant features, the Spearman correlation was performed between any two features. When highly correlated features were encountered (Spearman’s coefficient >0.9/<−0.9), only the feature with the lowest *p*-value in univariate analysis was retained.

The last step of feature reduction represented the multivariate logistic regression analysis using the least absolute shrinkage and selection operator (LASSO) with a ten -fold cross-validation.

Finally, five radiomic features were selected and combined into a Radiomic Signature, which was computed by taking a linear combination of the chosen features and weighting them based on their respective LASSO coefficients.

This three-step reduction technique was also performed in previous radiomic studies with favorable classification outcomes [18,19].

The diagnostic performance of the radiomic signature in both training and testing datasets was assessed by the Receiver operating characteristic (ROC) curve analysis.

The comparison between different areas under the ROC curve was performed using the DeLong test.

To analyze quantitative clinical and biological features, the independent-sample T or the Mann–Whitney U tests were employed, while for the assessment of categorical features, the exact Fisher test was utilized.

To identify independently associated features able to predict the malignancy of PGT, a binary logistic regression was performed using the enter method. The statistical significance value was set for *p* < 0.05.

The statistical analysis was conducted using the following software: SPSS Statistics for Windows, version 18.0 (SPSS Inc., Chicago, IL, USA), MedCalc version 14.8.1 (MedCalc Software, Mariakerke, Belgium), and R software version 3.6.3 (with the “glmnet” package).

**Figure 2:**
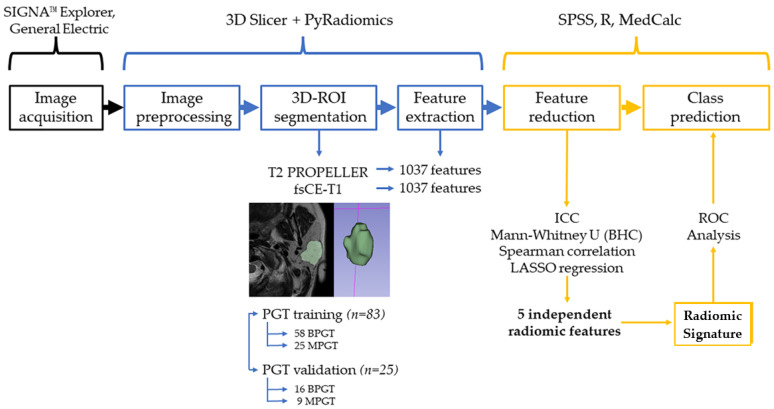
Radiomics workflow diagram. ROI = region of interest; PROPELLER = periodically rotated overlapping parallel lines with enhanced reconstruction; fsCE = fat-saturated contrast-enhanced; PGT = parotid gland tumors; BPGT = benign parotid gland tumor; MPGT = malignant parotid gland tumor; ICC = Intraclass Correlation Coefficient; BHC = Benjamini–Hochberg Correction; LASSO = Least Absolute Shrinkage and Selection Operator; ROC = Receiver-Operating Characteristic.

An overview of the radiomic workflow used in this study is offered in Figure 2.

## 3. Results

In this radiomic study, a total of 108 PGT were analyzed, which were divided into a training set (83 PGT: 58 benign, 25 malignant) and a testing set (25 PGT: 16 benign, 9 malignant). Tumor characteristics are summarized in Table 2 and the histopathological distribution is presented in Table 3.

In the training group, the patient’s age, maximum tumor size, tumor margin, T2-WI signal intensity compared to the parotid gland parenchyma, T2-WI signal intensity ratio (PGT signal intensity, divided by the masseter muscle signal intensity), and ADC value were significantly different between benign and malignant PGT.

MPGT appeared in older patients and presented greater dimensions than BPGT, predominant ill-defined margins, and hypointense signal on T2-WI. Furthermore, the T2-WI signal intensity ratio and the ADC values were lower for the MPGT in comparison to BPGT (2.85 vs. 3.96, *p* = 0.039; 0.855 vs. 1.205, *p* = 0.001, respectively).

Besides the age and the T2-WI intensity of PGT, all the parameters that proved to be statistically significant between the two study groups in the training set were also confirmed in the testing group.

### 3.1. Feature Selection and Radiomic Signature Construction in the Training Set

From each 3D segmentation of the PGT included in the training set, a total of 1037 radiomic features were extracted from T2-WI and another 1037 radiomic features from the fsCE-T1-WI, respectively. The intrareader agreement was tested and features with an ICC < 0.75 were excluded from further statistical analysis. Therefore, the features were reduced to 859 T2-WI features and 834 fsCE-T1-WI features.

**Figure 3:**
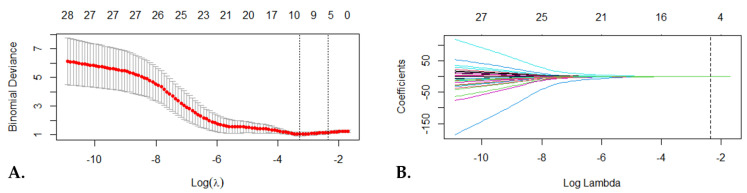
LASSO (least absolute shrinkage and selection operator) regression model. (**A**) Tuning parameter lambda (λ) selection using ten-fold cross-validation. The most favorable λ value was 0.045 and log (λ) = −3.08. The most favorable λ value according to the minimum criteria and 1 standard error of the minimum criteria are depicted with the dotted vertical lines. (**B**) The LASSO regression coefficients of the final 28 radiomics features and the most favorable λ (dotted vertical line).

To develop the radiomic signature, firstly, a univariate analysis was performed, and 67 T2-WI features and 50 fsCE-T1-WI features were found to be statistically significant between the two studied groups (with an adjusted *p*-value < 0.05 after Benjamini–Hochberg correction). Secondly, after performing Spearman’s correlation analysis, 28 non-redundant features were retained for further analysis (Supplementary File: Table S1, Figure S1). The final parameter reduction technique was the ten-fold cross-validated LASSO (least absolute shrinkage and selection operator) regression which revealed five final radiomic features (Figure 3). By linearly combining these five radiomic features using their corresponding LASSO coefficients (Table 4), the following radiomic signature formula was generated:

(1) $$Radiomic\;Signature=\sum_{x=0}^{5}C_{x}*\;R_{x}+I,$$

where Cx is the coefficient of the *x*th radiomic feature, R*x* the *x*th radiomic feature, and I the intercept.

### 3.2. The Performance of the Radiomic Signature in the Training Set

There was a significant difference in the radiomic signature value between BPGT and MPGT, −1.11 [−1.31, −0.71] versus −0.34 [−0.70, −0.15], *p* < 0.001.

**Figure 4:**
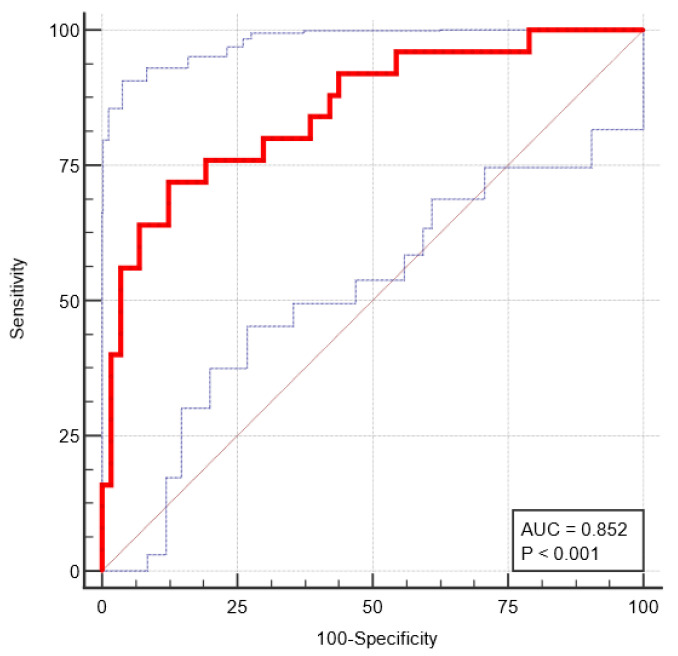
Receiver operating characteristic (ROC) curve of the radiomic signature for differentiating between BPGT and MPGT-training set (red line—ROC curve; thin dotted blue lines—ROC Confidence Interval).

In the training set, the radiomic signature predicted malignancy of PGT with an area under the curve (AUC) of 0.852, resulting in a sensitivity of 72% and a specificity of 87.7% for the cut-off value of >−0.614 (Figure 4).

The individual diagnostic performance of the five radiomic features was also assessed (Table 5). The AUC values varied between 0.668 and 0.747 and were lower than the one reached by the radiomic signature.

A clinical–radiological model was developed using a multivariate regression (Table 6) which included statistically significant features between BPGT and MPGT in the training group (age, maximum tumor size, margin, T2-WI signal intensity, T2-WI ratio, and ADC value). By adding the radiomic signature to the clinical–radiological model, a complex model was developed (Table 7). In the complex model, the radiomic signature and the ill-defined margin pattern were independently associated with malignancy of the PGT.

**Figure 5:**
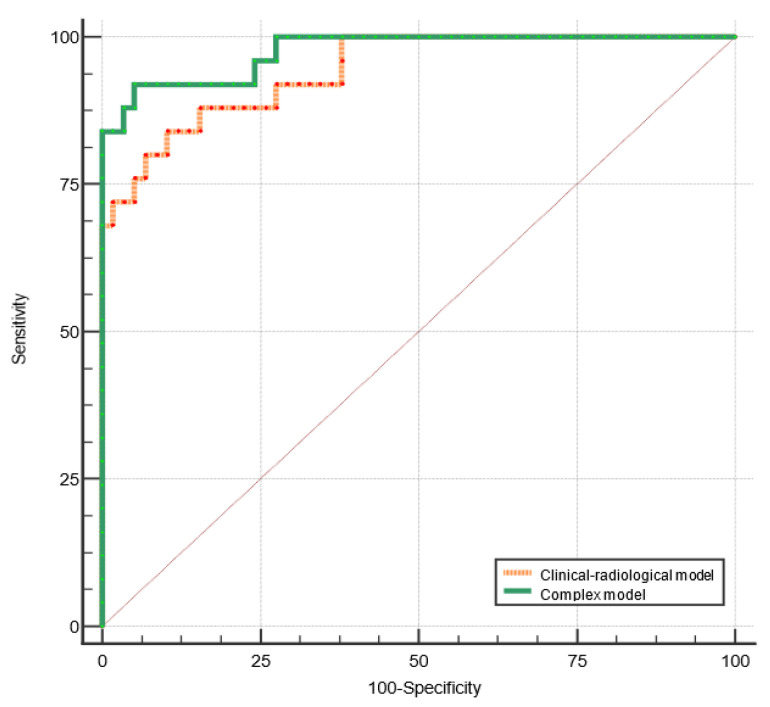
ROC curves of the clinical–radiological and complex models for differentiating between BPGT and MPGT.

Adding the radiomic signature to the first model slightly improved its performance for the differentiation between BPGT and MPGT: AUC = 0.943 (95% CI, 0.869 to 0.982) vs. AUC = 0.976 (95% CI, 0.916 to 0.997), *p* = 0.064, standard error = 0.02 (Figure 5).

**Figure 6:**
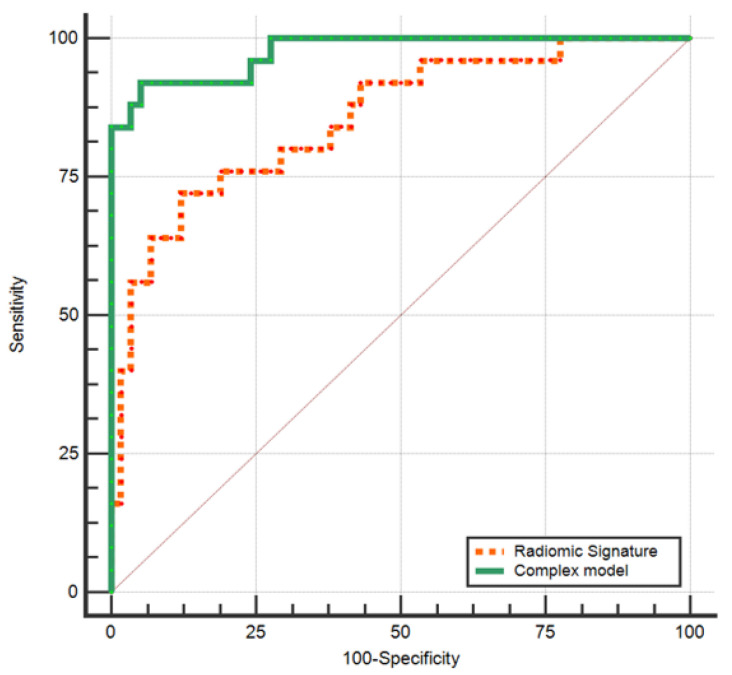
Comparison between ROC curves of the radiomic signature and the complex model for differentiating between BPGT and MPGT.

A statistically significant difference between the AUC obtained by the radiomic signature and the one reached by the complex model was found: the difference between areas = 0.123; standard error = 0.04; 95% confidence interval, 0.0306 to 0.212, *p* = 0.008 (Figure 6).

### 3.3. The Validation of the Radiomic Signature in the Testing Group

The radiomic signature of BPGT was significantly lower than MPGT (−0.859 vs. −0.527, *p* < 0.05) in the testing set as well (Table 8).

**Figure 7:**
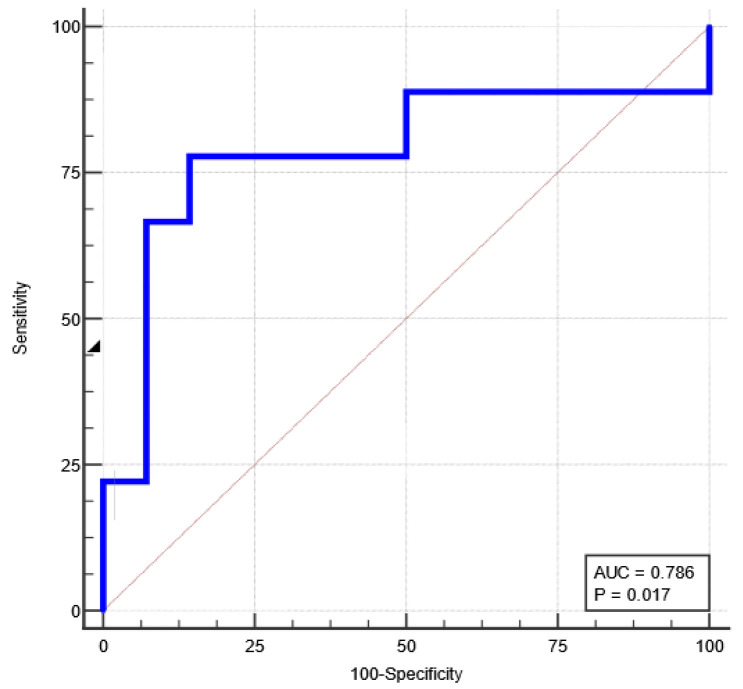
Receiver operating characteristic (ROC) curve (blue line) of the radiomic signature for differentiating between BPGT and MPGT in the testing set.

The overall diagnostic performance of the radiomic signature in differentiating between BPGT and MPGT was attested in the testing group, reaching an AUC of 0.786, when the optimal cut-off value was chosen according to the Jouden index analysis (Figure 7 and Table 9).

For the previous cut-off value of >−0.614 (established in the training group), in the validation group, there 66.7% sensitivity and 81.3% specificity were recorded.

## 4. Discussion

The current study assessed the performance of MRI-based radiomic features to discriminate between benign and malignant PGT using a 3D segmentation approach on T2-WI and fsCE-T1-WI sequences.

Radiomics represents a non-invasive postprocessing imaging technique that implies extracting numerous quantitative features from digital images, thereby converting them into mineable high-dimensional data which could predict changes at a cellular, metabolic, or genetic level [11,20]. Recently, there has been an increasing interest in the radiomics of salivary gland imaging, mainly addressing the following three topics: differentiating between different PGT types, predicting xerostomia after radiation therapy in the head-neck region, or assessing PG parenchymal changes in diffuse inflammatory diseases, such as primary Sjogren’s Syndrome [21].

The constructed radiomic signature in this study includes first-order radiomic features derived from the histogram, and second-order radiomic features from the gray-level co-occurrence matrix (GLCM) and gray-level size zone matrix (GLSZM). First-order features provide information about the voxels’ intensity distribution inside a region or volume of interest, regardless of any spatial relationships between each other, while second-order features are computed from various matrices to describe the spatial arrangement and interaction between voxels [22]. GLCM assesses the spatial relationships between individual voxels with different gray-level values, while GLSZM considers the size and frequency of connected regions of voxels with the same intensity value within a given region/volume of interest in a medical image [23,24].

The feature extraction process was performed from both unfiltered and filtered images. As a pre-processing step, to all images, we applied Laplacian of Gaussian filters, which highlight regions of rapid change (for instance, edge detection) and wavelet filters, which separate information with high and low spatial frequency [25]. The majority of the radiomic features included in the final radiomic signature were extracted from images with wavelet filters, which proved to be especially useful in imaging denoising [26].

Regarding the PGT segmentation, a volume of interest was delineated in the three-dimensional (3D) approach, as we considered it would provide representative information more reliable for entire tumor characterization and heterogeneity assessment in comparison to a 2D segmentation.

The chosen MRI sequences for segmentation were T2-WI acquired with the PROPELLER technique and fsCE-T1-WI. By using PROPELLER, the image quality increases, and any potential motion artifacts are diminished [27]. Contrast-enhanced imaging is often used in radiomic studies as it provides information regarding tumor vascularity and heterogeneity that would not be discernible without the use of contrast material [15].

The results obtained in this study demonstrate that the proposed MRI-based radiomic signature, obtained from the combination of five radiomic features extracted from two standard sequences, achieved a promising performance in differentiating benign from malignant PGT. The AUC of the radiomic signature in the training group was 0.852, higher than the AUC of each radiomic feature individually, which ranged between 0.668–0.747. In the testing dataset, the radiomic signature maintained a good diagnostic performance, with an AUC of 0.786.

There are several studies performed so far that assessed the ability of MRI radiomic features to differentiate MPGT from BPGT using either features extracted from one sequence alone or multiple sequences combined. The reported diagnostic performances of the obtained radiomic models are variable.

The best radiomic model proposed by Zheng et al. was built using a combination of T1-WI-logarithm and fsT2-WI-exponential features and reached an average AUC of 0.846 for differentiating benign from malignant SGT. This study, however, included not only PGT, as in our study, but also tumors of the submandibular and sublingual glands [28]. By combining radiomic features extracted from manual segmentations of PGT on T1- and T2-WI, Vernuccio et al. obtained a radiomic model that presented an AUC of 0.927, with high specificity (93.4%), albeit at a cost of low sensitivity (57.2%) in the diagnosis of PGT malignancy [29].

Another study used Linear Discriminant Analysis (LDA) and Support Vector Machine (SVM) classifiers to discriminate between BPGT and MPGT on T2-WI, ADC map, and DCE-MRI, and the reported AUC for each sequence was 93.3%, 100%, 99.2% for LDA, and 100%, 100%, 100% for SMV, respectively. When the classifiers were trained using features from all three sequences combined, the AUC reached 100% for both LDA and SMV [30].

Using only the conventional T2-WI, SVM classifier training was performed using five non-redundant discriminative features between BPGT and MPGT, and all feature subsets were tested as well. The radiomic signature resulting after combining the gray-level mean, skewness, and autocorrelation values performed the best in classifying PGT, yielding a high specificity (88.5%), but low sensitivity and accuracy (29.4%, 0.594, respectively) [31].

One study proposed a mixed model that combined four parameters: the 25th percentile extracted from the ADC map, the 10th percentile extracted from T2-WI, and the type of contrast enhancement and margins. The model’s accuracy in differentiating BPGT from MPGT was 80.4% in the training cohort and 89.2% in the validation cohort [32].

A multisequence combined radiomic model presented a higher differentiation performance (BPGT vs MPGT, AUC = 0.863) than the single- or double-sequence radiomic models (AUC between 0.792–0.855), while the radiomic nomogram reached the highest AUC of 0.907 [33]. Liu et al. also proved that adding 2D and/or 3D radiomic features extracted from T1- and T2-WI to clinical and radiological data improved the diagnostic performance in differentiating BPGT from MPGT (0.85 AUC for the complex model versus 0.72 AUC for the clinical reference model) [34].

Similarly, to provide a more holistic model, we have also performed a multivariate analysis using clinical data, MRI features, and the radiomic signature. This integrated approach improved the diagnostic performance in differentiating BPGT from MPGT, in comparison to the radiomic signature alone.

Radiomic imaging is under constant development and recent studies aim to propose new and improved classification algorithms and radiomic signatures. However, there is still a need to prove how radiomic features can systematically outperform other competitors, ideally across multiple datasets.

Using multiparametric MRI in assessing PGT has already demonstrated promising results, currently representing the first-line imaging method in assessing this pathology. According to Zeng et al., using conventional MRI and DWI combined reached 91% diagnostic accuracy in differentiating between MPGT and BPGT (82% sensitivity and 94% specificity), while adding DCE MRI to the assessment boosted the accuracy to 96% [35]. This was confirmed in another study which showed that assessing the tumor shape and capsule presence on conventional MRI sequences in conjunction with functional MRI features (such as ADC and TIC patterns) demonstrated great performance: 91.2% accuracy in diagnosing parotid gland lesions, i.e., higher than the individual diagnostic performance of each MRI sequence [36].

Our proposed radiomic signature reached a sensitivity of 72% and a specificity of 87.7% in differentiating between BPGT and MPGT and the AUC was 0.852. When clinical–radiological data were added and a complex model was built, the AUC increased to 0.976. This supports the idea that radiomics present great diagnostic potential when used in addition to other imaging features and not just as an individual diagnostic tool [21].

However, the current study presents several limitations. Firstly, there was a relatively low number of observations, particularly in the MPGT group. This is due mainly to a generally higher incidence of benign PGT (approximately 80%) [3]. Secondly, although we were able to split the acquired observations into a training and testing dataset, the obtained radiomic signature lacks external validation on a new data set from a different institution, which would have increased the signature’s generalizability. The logistic regressions were only performed on the training dataset and were not assessed in the testing dataset due to the low number of cases assigned in the latter, which would not have allowed a fair regression analysis with statistical significance.

MR images are known to be subject to increased variations in signal intensity between examinations, which is a main problem in radiomics that requires special consideration [37,38]. As this study was monocentric, all patients were examined in the radiology department using a standardized MRI head-neck protocol. However, there were still some variations in the acquisition parameters (TE, TR) and consequently, before PGT segmentation and feature extraction, all MR images underwent several pre-processing techniques to obtain a homogenous set of images.

Another important aspect is related to the ability of many texture analysis programs to extract a high number of radiomic features, which often exceeds the number of samples and therefore increases the risk of overfitting [11,39]. To counteract this aspect, we have performed several feature reduction techniques to exclude redundant features. We have also performed corrections for multiple testing, and *p*-values were adjusted using a false discovery rate method such as the Benjamini–Hochberg correction [40].

Some authors report an interobserver variability regarding the segmentation of the PG, which might impact the value of the extracted radiomic feature [41]. Unfortunately, this confounder effect was not assessed in the current study, and only the intraobserver variability was tested.

Finally, the current study only assessed radiomic features extracted from two conventional anatomical MRI sequences and did not assess other sequences such as diffusion or perfusion-weighted imaging.

## 5. Conclusions

The current study proved that radiomic features extracted from MR images and the proposed radiomic signature are able to discriminate between benign and malignant parotid gland tumors. An integrated approach using clinical, radiological, and radiomic features achieved a better diagnostic performance than the radiomic signature alone, suggesting the current adjuvant role of radiomics as a diagnostic tool. Nevertheless, the validation of the proposed radiomic model in larger, multicentric studies, is warranted.

## Figures and Tables

**Table 1:** MRI sequences specifications.

MRI Parameter	T2-WI	fsCE-T1-WI
TE (ms) *	75 [62–92]	12 [8.9–15.6]
TR (ms) *	5450 [3540–8290]	680 [610–750]
Matrix (mm)	384 × 384	300 × 300
Flip angle	160	160
Slice thickness (mm)	3	3
Slice gap (mm)	3	3

* median (interquartile range).

**Table 2:** Clinical and radiological characteristics of the benign and malignant PGT in the training and testing sets.

Feature		Training Set (n = 83)		*p*	Testing Set (n = 25)		*p*
		Benign PGT (n = 58)	Malignant PGT (n = 25)		Benign PGT (n = 16)	Malignant PGT (n = 9)	
Age (years)		49.6 ± 14.7	58.1 ± 13.4	0.014	54.2 ± 12.12	63.22 ± 14.87	0.115
Sex	Male	25 (43.1)	10 (40)	0.794	8 (50)	4 (44.4)	0.793
	Female	33 (56.9)	15 (60)		8 (50)	5 (55.6)	
Maximum size (mm)		26 [19, 32]	35 [25.7, 48.2]	0.011	19.5 [13, 24.5]	31 [21.5, 39]	0.046
Location	Superficial	35 (60.3)	10 (40)	0.14	13 (81.2)	8 (88.9)	0.513
	Deep	6 (10.3)	2 (8)		1 (6.3)	1 (11.1)	
	Both	17 (29.3)	13 (52)		2 (12.5)	0 (0)	
Side	Left	30 (51.7)	13 (52)	0.8	8 (50)	2 (22.2)	0.182
	Right	28 (48.3)	12 (48)		8 (50)	7 (77.8)	
Margin	Smooth	56 (96.6)	7 (28)	<0.001	14 (87.5)	2 (22.2)	0.001
	Ill-defined	2 (3.4)	18 (72)		2 (12.5)	7 (77.8)	
Cystic/necrotic areas	Present	36 (62.1)	16 (64)	0.755	12 (75)	5 (55.6)	0.327
	Absent	22 (37.9)	9 (36)		4 (24)	4 (44.4)	
T1-WI hyperintense spots	Present	14 (24.1)	2 (8)	0.089	8 (50)	7 (77.8)	0.228
	Absent	44 (75.9)	23 (92)		8 (50)	2 (22.2)	
T2-WI signal (vs. parotid)	Hypointense	16 (29.6)	17 (68)	<0.001	6 (37.5)	6 (66.7)	0.169
	Hyperintense	42 (72.4)	8 (32)		10 (62.5)	3 (33.3)	
T1-WI signal (vs. muscle)	Hypointense	49 (84.5)	24 (96)	0.141	14 (87.5)	9 (100)	0.278
	Hyperintense	9 (15.5)	1 (4)		2 (12.5)	0 (0)	
CE pattern	Homogenous	19 (32.7)	5 (20)	0.752	3 (18.7)	4 (44.4)	0.244
	Heterogenous	39 (67.2)	20 (80)		13 (81.3)	5 (55.6)	
T2-WI SI Ratio *		3.96 [2.46, 6.09]	2.85 [2.36, 3.66]	0.039	3.82 [2.51, 5.36]	2.56 [2.13, 2.88]	0.047
T1-WI SI Ratio *		1.14 [1, 1.27]	1.17 [1.08, 1.30]	0.605	1.23 [1.08, 1.39]	1.26 [1.10, 1.74]	0.428
fsCE-T1-WI SI Ratio *		1.61 [1.40, 2.18]	1.50 [1.40, 1.91]	0.461	1.33 [1.16, 1.95]	1.15 [1.11, 1.39]	0.212
ADC (10^−3^ mm^2^/s)		1.205	0.855	0.001	1.176	0.875	0.019
		[0.910, 1.800]	[0.728, 1.185]		[0.865, 1.459]	[0.670, 0.908]	

CE = contrast enhancement; SI = signal intensity; fs = fat-saturated; ADC = apparent diffusion coefficient; PGT = parotid gland tumor; *p* = statistical significance level. The results are presented as mean ± standard deviation, median and [interquartile range], or percentage (%). * Parotid gland tumor signal intensity/Masseter muscle signal intensity.

**Table 3:** Histopathology of parotid gland tumors.

	Training Set		Testing Set	
	Benign (n = 58)	Malignant (n = 25)	Benign (n = 16)	Malignant (n = 9)
Tumor histology	Pleomorphic adenoma 28	Mucoepidermoid cc 3	Pleomorphic adenoma 6	Acinic cell cc 2
	Warthin tumor 24	Salivary duct cc 3	Warthin tumor 8	Salivary duct cc 2
	Basal cell adenoma 3	Adenoid cystic cc 3	Basal cell adenoma 1	Metastatic cc 2
	Parotid gland cyst 2	Acinic cell cc 2	Oncocytoma 1	Lymphoma 3
	Reactive lymph node 1	Lymphoma 7		
		Metastatic cc 3		
		Basal cell cc 2		
		Squamous cell cc 1		
		Undifferentiated sarcoma 1		

**Table 4:** Radiomic feature selection results after LASSO regression.

MRI Sequence	Radiomic Feature	Radiomic Group	Associated Filter	Coefficient
fsCE-T1-WI	SizeZoneNonUniformityNormalized	Texture (glszm)	original	−0.865
fsCE-T1-WI	Skewness	First order	LoG filter (5 mm)	0.09
T2-WI	RootMeanSquared	First order	wavelet-HLL	0.136
T2-WI	Imc2	Texture (glcm)	wavelet-LLH	−0.167
T2-WI	Correlation	Texture (glcm)	wavelet-LHL	−0.296
	Intercept			−0.865

fsCE = fat-saturated with contrast enhancement; glszm = gray-level size zone matrix; glcm = gray-level co-occurrence matrix; LoG = Laplacian of Gaussian.

**Table 5:** Individual diagnostic performance of the final selected radiomic features.

Radiomic Feature	AUC (95% CI)	Cut-Off	Se (95% CI)	Sp (95% CI)	+LR (95% CI)	−LR (95% CI)	*p*
SizeZoneNonUniformityNormalized	0.668	≤−0.509	60	73.68	2.28	0.54	0.01
	(0.555–0.768)		(38.7–78.9)	(60.3–84.5)	(1.33–3.91)	(0.33–0.90)	
Skewness	0.695	>0.135	80	63.16	2.17	0.32	0.001
	(0.584–0.792)		(59.3–93.2)	(49.3–75.6)	(1.47–3.21)	(0.14–0.71)	
RootMeanSquared	0.678	>0.329	48	82.46	2.74	0.63	0.006
	(0.566–0.777)		(27.8–68.7)	(70.1–91.3)	(1.37–5.48)	(0.42–0.94)	
Imc2	0.74	≤−0.361	72	70.18	2.41	0.4	<0.001
	(0.631–0.830)		(50.6–87.9)	(56.6–81.6)	(1.51–3.85)	(0.21–0.77)	
Correlation	0.747	≤−0.211	76	70.18	2.55	0.34	<0.001
	(0.639–0.836)		(54.9–90.6)	(56.6–81.6)	(1.62–4.02)	(0.17–0.70)	

The 95% confidence interval (CI) values are shown in parentheses. AUC = area under curve; Se = sensitivity; Sp = specificity; +LR = positive likelihood ratio; −LR = negative likelihood ratio; *p* = statistical significance level.

**Table 6:** Multivariate logistic regression analysis for the PGT malignancy prediction—clinical–radiological model.

Variable	Coefficient	Std. Error	*p*	Odds Ratio
Patient’s age	0.083	0.047	0.076	1.087
Maximum diameter	−0.016	0.047	0.736	0.984
Margin = “ill-defined”	5.649	1.488	<0.001	28.923
T2-WI = “hypointense”	2.520	1.491	0.091	12.435
T2-WI Ratio	0.030	0.387	0.937	1.030
ADC	−0.527	1.285	0.681	0.590
Constant	−7.558	4.341	0.081	

Std. Error = standard error; *p* = statistical significance level.

**Table 7:** Multivariate logistic regression analysis for the PGT malignancy prediction—complex model.

Variable	Coefficient	Std. Error	*p*	Odds Ratio
Patient’s age	0.087	0.046	0.059	1.09
Maximum diameter	−0.029	0.052	0.582	0.97
Margin = “ill-defined”	7.277	2.332	0.001	29.21
T2-WI = “hypointense”	1.501	1.544	0.330	4.48
T2-WI Ratio	−0.146	0.468	0.753	0.86
ADC	−1.159	1.822	0.524	0.31
Radiomic Signature	5.307	2.467	0.031	22.43
Constant	−2.410	4.318	0.576	

Std. Error = standard error; *p* = statistical significance level.

**Table 8:** Radiomic signature values of benign and malignant parotid gland tumors in the training and testing datasets.

Radiomic Signature	Median	Q1	Q3	*p*
Training set				
Benign	−1.11	−1.31	−0.71	<0.001
Malignant	−0.34	−0.7	−0.15	
Testing set				
Benign	−0.859	−1.435	−0.785	0.023
Malignant	−0.527	−0.787	−0.36	

Q1 = first quartile; Q3 = third quartile; *p* = statistical significance level.

**Table 9:** Diagnostic performance of the radiomic signature in the training and testing datasets.

Radiomic Signature	AUC (95% CI)	Cut-Off	Se (95% CI)	Sp (95% CI)	+LR (95% CI)	−LR (95% CI)	*p*
Training set	0.852	>−0.614	72	87.72	5.86	0.32	<0.0001
	0.756–0.921		50.6–87.9	76.3–94.9	2.81–12.23	0.17–0.60	
Testing set	0.786	>−0.774	77.78	85.71	5.44	0.26	0.017
	0.566–0.927		40.0–97.2	57.2–98.2	1.44–20.58	0.075–0.90	

The 95% confidence interval (CI) values are shown in parentheses. AUC = area under curve; Se = sensitivity; Sp = specificity; +LR = positive likelihood ratio; −LR = negative likelihood ratio; *p* = statistical significance level.

## Data Availability

The data are available only by request.

## Supplementary Materials

The following supporting information can be downloaded at: https://www.mdpi.com/article/10.3390/cancers15133319/s1, Table S1: List of the selected radiomic features able to discriminate between benign and malignant PGT, that were included in the LASSO regression analysis. Figure S1: Correlogram of the final selected radiomic features (n = 28) using the Spearman’s correlation.
