# Supplementary material for: The Role of an MRI-Based Radiomic Signature in Predicting Malignancy of Parotid Gland Tumors
Source: Cancers (Basel). 2023 Jun 23;15(13):3319. doi: 10.3390/cancers15133319 (PMC10340186; doi:10.3390/cancers15133319)
Supplement: Supplementary file 1 [file cancers-15-03319-s001.zip › cancers-2375157-supplementary-updated.pdf]

cancers MDPI

## Supplimentary File

**Table S1.** List of the selected radiomic features able to discriminate between benign and malignant PGT, that were included in the LASSO regression analysis.

| MRI Sequence | Radiomic Feature | Radiomic Group | Associated Filter | *p*-value* |
|---|---|---|---|---|
| T2-WI | Contrast | NGTDM | LoG filter (3 mm) | 0.026 |
| T2-WI | DifferenceEntropy | GLCM | LoG filter (5 mm) | 0.010 |
| T2-WI | LongRunEmphasis | GLRLM | LoG filter (5 mm) | 0.024 |
| T2-WI | Imc1 | GLCM | wavelet-LLH | 0.004 |
| T2-WI | Strength | NGTDM | wavelet-LHL | 0.032 |
| T2-WI | Contrast | GLCM | wavelet-LHH | 0.003 |
| T2-WI | Imc2 | GLCM | wavelet-LHH | 0.044 |
| T2-WI | DependenceVariance | GLDM | wavelet-LHH | 0.042 |
| T2-WI | Mean | firstorder | wavelet-HLL | 0.026 |
| T2-WI | RootMeanSquared | firstorder | wavelet-HLL | 0.017 |
| T2-WI | RootMeanSquared | firstorder | wavelet-HLH | 0.039 |
| T2-WI | GrayLevelNonUniformityNormalized | GLSZM | wavelet-HLH | 0.029 |
| T2-WI | Mean | firstorder | wavelet-LLL | 0.012 |
| T2-WI | Busyness | NGTDM | wavelet-LLL | 0.015 |
| T2-WI | Imc2 | GLCM | wavelet-LLH | 0.002 |
| T2-WI | Correlation | GLCM | wavelet-LHL | <0.001 |
| T2-WI | ClusterTendency | GLCM | wavelet-LLH | 0.005 |
| T2-WI | SizeZoneNonUniformityNormalized | GLSZM | wavelet-LLH | 0.017 |
| T2-WI | SumAverage | GLCM | wavelet-LLL | 0.029 |
| T2-WI | GrayLevelVariance | GLSZM | wavelet-LLL | 0.039 |
| fsCE-T1-WI | SizeZoneNonUniformityNormalized | GLSZM | original | 0.014 |
| fsCE-T1-WI | SmallDependenceEmphasis | GLDM | LoG filter (5 mm) | 0.049 |
| fsCE-T1-WI | SizeZoneNonUniformityNormalized | GLSZM | wavelet-LHL | 0.014 |
| fsCE-T1-WI | SmallAreaEmphasis | GLSZM | wavelet-LHL | 0.021 |
| fsCE-T1-WI | HighGrayLevelRunEmphasis | GLRLM | wavelet-LHH | 0.003 |
| fsCE-T1-WI | Skewness | firstorder | wavelet-HLL | 0.010 |
| fsCE-T1-WI | HighGrayLevelRunEmphasis | GLRLM | wavelet-HLH | <0.001 |
| fsCE-T1-WI | Skewness | firstorder | wavelet-HHH | 0.042 |

fsCE=fat-saturated with contrast enhancement; ngtdm = neighbouring gray tone difference matrix; glcm = gray-level co-occurrence matrix; glrlm = gray-level run length matrix; gldm = gray-level dependence matrix; glszm = gray-level size zone matrix; LoG=Laplacian of Gaussian; *p value in the univariate analysis using Mann-Whitney U test and the Benjamini Hochberg correction for multiple tests.

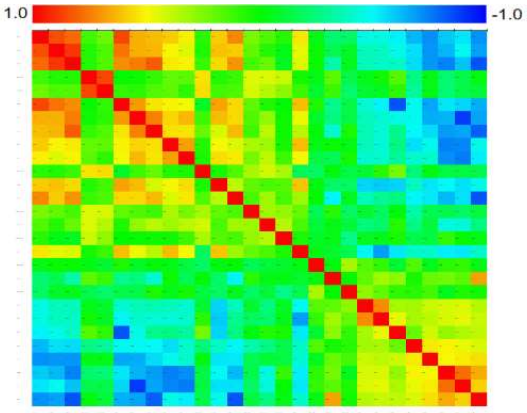


**Figure S1.** Correlogram of the final selected radiomic features (n=28) using the Spearman's correlation.
